# Intradermal acupuncture for primary dysmenorrhea: A protocol of systematic review and meta-analysis of randomized clinical trials

**DOI:** 10.1097/MD.0000000000022188

**Published:** 2020-09-11

**Authors:** Donghan Xu, Lizhen Wang, Lu Deng, Yehao Luo, Yan Wei, Peiyu Yan

**Affiliations:** a Macau University of Science and Technology, College of Traditional Chinese Medicine, Macau; b Hospital of Chengdu University of Traditional Chinese Medicine, Chengdu, Sichuan Province; c Zhuang College of Medicine, Guangxi University of Traditional Chinese Medicine, Nanning; d Zhuhai Hospital of Integrated Traditional Chinese and Western Medicine, Acupuncture Department, Zhuhai, China.

**Keywords:** intradermal acupuncture, meta analysis and systematic review, primary dysmenorrhea, protocol

## Abstract

**Background:**

Primary dysmenorrhea (PD) is one of the common gynecological diseases, the incidence of PD is on the rise and young women are more likely to have it, which seriously affects women's physical, mental health and work life. Intradermal acupuncture is effective in treating PD. However, due to the lack of evidence, there is no specific method or suggestion, so it is necessary to carry out systematic evaluation on intradermal acupuncture and provide effective evidence for further research.

**Methods:**

We will search the following electronic databases from their inception to July 2020: Electronic database includes PubMed, Embase, Cochrane Library, Chinese Biomedical Database WangFang, VIP medicine information, and CNKI (China National Knowledge Infrastructure). Primary outcomes: the overall effective rate, VAS score. Secondary outcomes: blood serum estradiol (E2), progesterone (P), prostaglandin F2α (PGF-2α), adverse events Data will be extracted by 2 researchers independently, risk of bias of the meta-analysis will be evaluated based on the Cochrane Handbook for Systematic Reviews of Interventions. All data analysis will be conducted by data statistics software Review Manager V.5.3. and Stata V.12.0.

**Results:**

The results of this study will systematically evaluate the effectiveness and safety of intradermal acupuncture in the treatment of primary dysmenorrhea.

**Conclusion:**

The systematic review of this study will summarize the currently published evidence of intradermal acupuncture therapy for primary dysmenorrhea to further guide its promotion and application.

## 1 Introduction

Primary dysmenorrhea (PD) is the lower abdomen pain that occurs periodically before or during menstruation in the absence of any organic lesions.^[1]^ PD tends to occur in adolescent girls and unmarried young women,^[2,3]^ the incidence is 48% to 89%.^[4,5]^ The pain generally lasts for 8 to 72 hours, and is most severe on the first and second days of menstruation. Severe patients are often accompanied by nausea, vomiting and other symptoms.^[6]^ Unfortunately, the incidence of PD is on the rise,^[7]^ which seriously affects women's physical, mental health and work life.^[8]^

Western medicine treatment of primary dysmenorrhea is mostly based on NSAIDs, contraceptives, calcium channel blockers, vitamins, etc. through investigation, it is found that the above drugs are used extensively, and the side effects are obviously, especially gastrointestinal reaction and water sodium retention.^[9]^ The medication compliance of PD patients is greatly reduced, so it is necessary to seek a safe, reliable and effective treatment method.

In recent years, acupuncture has been widely used in clinical and experimental studies of PD, and its effectiveness has been fully proved. As a form of acupuncture, the intradermal acupuncture has been used to improve symptoms in PD patients, but its effectiveness and safety have not yet reached a definitive conclusion. Therefore, this research intends to adopt the method of system valuation and meta analysis of the intradermal acupuncture for PD to evaluate the efficacy and safety.

## 2 Methods

### 2.1 Study registration

The protocol of the systematic review has been registered.

Registration: OSF Preregisration.2020, August 12. https://osf.io/cxb5w. This systematic review protocol will be conducted and reported strictly according to Preferred Reporting Items for Systematic Reviews and Meta-Analyses (PRISMA)^[10]^ statement guidelines, and the important protocol amendments will be documented in the full review.

### 2.2 Inclusion and exclusion criteria for study selection

#### 2.2.1 Inclusion criteria

Inclusion criteria are all randomized controlled trials (RCTs), which main treatment of PD is intradermal acupuncture. The language of the trials to be included only Chinese or English.

#### 2.2.2 Exclusion criteria

Following studies will be excluded:

1. Repeated publications
2. Review of literature and cases
3. Animal studies
4. Incomplete literature
5. Non-randomized controlled trials

### 2.3 Types of participants

The types of subjects included patients diagnosed with primary dysmenorrhea, regardless of their degree and possible complications. All patients were treated with intradermal acupuncture. Due to the different pathogenesis and mechanism, we will exclude patients with secondary dysmenorrhea caused by endometriosis and adenomyosis.

### 2.4 Interventions and controls

Interventions included treatment with intradermal acupuncture. The control group only received conventional western medicine treatment. The routine treatment of each RCT may not be identical, but the use of intradermal acupuncture is the only difference between intervention and control.

### 2.5 Types of outcome measures

#### 2.5.1 Main outcomes

1. The overall effective rate;
2. VAS score.

#### 2.5.2 Additional outcomes

1. Progesterone (P);
2. Blood serum estradiol (E2);
3. Prostaglandin F2α (PGF-2α);
4. Adverse events.

### 2.6 Search methods

#### 2.6.1 Search resources

**Figure 1:**
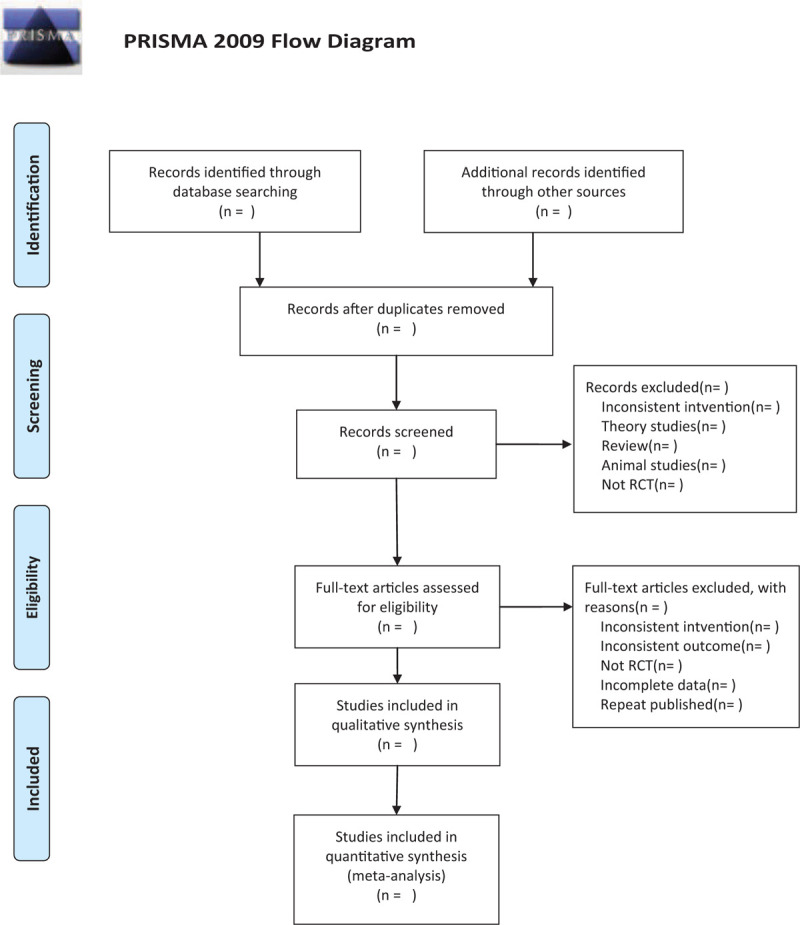
The research flowchart. This figure shows the identification, screening, eligibility and included when we searching articles.

We will search the following electronic databases from their inception to July 2020: Electronic database includes PubMed, Embase, Cochrane Library, Chinese Biomedical Database WangFang, VIP medicine information, and CNKI (China National Knowledge Infrastructure) (Fig. 1).

#### 2.6.2 Search strategies

The following MeSH terms and their combinations will be searched: (1) intradermal acupuncture; (2) RCT OR RCTs; (3) Primary dysmenorrhea. The search strategy for PubMed is shown in Table 1. Other electronic data bases will be used the same strategy.

**Table 1:**
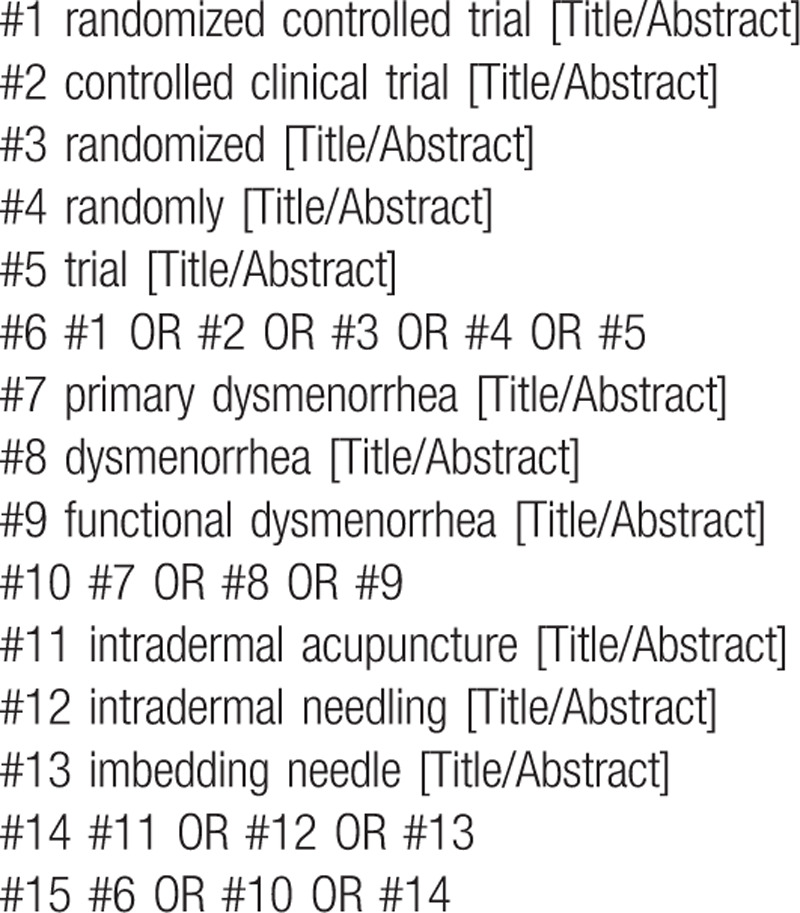
Search strategy in PubMed database.

### 2.7 Data collection and analysis

#### 2.7.1 Studies selection

There will be 2 researchers (DX and LW) carry out the selection of research literature independently using NoteExpress software. We will first make the preliminary selection by screening titles and abstracts. Secondly, we will download full text of the relevant studies for further selection according to the inclusion criteria. If there is any different opinion, 2 researchers will discuss and reach an agreement. If a consensus could not be reached, there will be a third researcher (PY) who make the final decision. The details of selection process will be displayed in the PRISMA flow chart.

#### 2.7.2 Data extraction

Two researchers (LD and YL) will read all the included text in full, and independently extract the following information: (1) general information, including trial name and registration information; (2) trial characteristic, including trial design, location, setting, and inclusion/exclusion criteria;(3)the characteristics of the participants, including age, race/ethnicity, course of illness, etc.(4)details of intervention, including acupoints, time of intervention, course of treatment, time of single treatment, etc;(5)details of comparison interventions; (6) outcomes as described under type of outcome measure section. If we could not reach an agreement, a third researcher (YW) would make the final decision. One researcher (LD) would contact the corresponding author by telephone or e-mail for more information when the reported data were insufficient or ambiguous.

#### 2.7.3 Assessment of risk of bias

All the included studies will be evaluated based on the guidelines of Cochrane Handbook for Systematic Reviews of Interventions.^[11]^ The quality of each trial will categorized into “low”, “unclear”, or “high” risk of bias according to the following items: adequacy of generation of the allocation sequence, allocation concealment, blinding of participants and personal, blinding of outcome assessors, incomplete outcome data, selected reporting the results and other sources of bias (such as comparable baseline characteristic, inclusion and exclusion criteria).

#### 2.7.4 Assessment of reporting biases

Reporting biases and small-study effects will be detected by funnel plot and Egger's test if there are 10 more studies included in this Meta- analysis. For Egger's test, *P* value of <.10 was considered to indicate the exist of reporting biases and small study effects.

#### 2.7.5 Data analysis

We used Revman 5.3 software provided by the Cochrane collaboration to analyze the data. Binary outcomes will be summarized using risk ratio (RR) with 95% confidence interval (CI) for relative effect. Continuous outcomes will be summarized by using weighted mean difference (WMD) with 95% CI. We will use random-effect model (REM) for meta-analysis in this review according to research recommendations.^[12]^

Statistical heterogeneity will be assessed by *X*^2^ and *I*^2^ statistical tests. Where *P* value ≥.1 and *I*^2^ ≤50%, there is no obvious statistical heterogeneity among the studies. On the contrary, where *P* value <.1 or *I*^2^ >50% indicates a considerable heterogeneity. Meta-analysis will be performed when the statistical heterogeneity is acceptable (*P* value ≥.1 and *I*^2^ ≤50%), otherwise, subgroup analysis will be applied to explore the influence of potential factors on the outcome measures. We will conduct sensitivity analyses by omitting studies one by one in order to probe the impact of an individual study. If a meta analysis cannot be performed, we will conduct descriptive analysis instead.

#### 2.7.6 Patient and public involvement

This is a meta-analysis study based on previously published data, so patient and public involvement will not be included in this study.

#### 2.7.7 Ethics and dissemination

Ethical approval will not be required as this is a protocol for systematic review and meta-analysis. The findings of this study will be disseminated to a peer-reviewed journal and presented at a relevant conference.

#### 2.7.8 Evidence assessed

The quality of evidence for this study will be assessed by “Grades of Recommendations Assessment, Development and Evaluation (GRADE)” standard established by the World Health Organization and international organizations.^[13]^ To achieve transparency and simplification, the quality of evidence is divided into 4 levels in GRADE system: high, medium, low and very low. We will employ GRADE profiler 3.2 for analysis.^[14]^

## 3 Discussion

Currently most western medicine is used to treat PD, although the effect is quick, it can only temporarily relieve the pain and is prone to relapse after withdrawal of the drug.^[15,16]^ The treatment of PD by intradermal acupuncture is a kind of acupuncture method, which can be used to treat primary dysmenorrhea by inserting the sterile map pin type intradermal acupuncture into the subcutaneous acupoints on the body surface, and then burying the needle in the body for a long time, and giving the skin a small amount of stimulation for a long time to regulate the Qi and blood of the meridians. The evidence has emerged that physical acupuncture is helpful in the treatment of PD, but extensive data on intradermal acupencture is still lacking. Therefore, intradermal acupuncture in the treatment of PD needs to be further clarified.

This systematic review will evaluate published RCTs evidence for the effectiveness and safety of intradermal acupuncture for PD. This study has several strengths, it may assist clinicians and patient treatment for primary dysmenorrhea with guidelines. Clinical research will be conducted based on this systematic review protocol. Intradermal acupuncture is a painless treatment method which does not directly stimulate the nerve endings of pain sensation, so it does not produce pain. The patients compliance greatly improved because of this advantage. In addition, compared with common acupuncture, intradermal acupuncture has thin and short body which can get more security. The long-term embedding of intradermal acupuncture in the subcutaneous and myofascial layers can produce the electrochemical effect of acupuncture to the greatest extent, and the long-term stimulation can regulate the function of botanic nerves, promote blood circulation, and achieve better curative effect.^[17,18]^

In summary, this review will be the first to evaluate the impact of intradermal acupuncture for PD. On the basis of this review, better methods for treating PD may be established and provide a reliable basis for its wide application.

## Author contributions

**Conceptualization:** Donghan Xu, Lizhen Wang, Peiyu Yan.

**Data curation:** Lu Deng, Yehao Luo.

**Formal analysis:** Donghan Xu, Lizhen Wang.

**Methodology:** Donghan Xu, Lizhen Wang, Peiyu Yan.

**Project administration:** Peiyu Yan.

**Resources:** Donghan Xu, Lizhen Wang, Yehao Luo.

**Software:** Donghan Xu, Lizhen Wang.

**Supervision:** Yan Wei.

**Writing – original draft:** Donghan Xu.

**Writing – review & editing:** Donghan Xu.
